# Relative impact of COPD and comorbidities on generic health-related quality of life: a pooled analysis of the COSYCONET patient cohort and control subjects from the KORA and SHIP studies

**DOI:** 10.1186/s12931-016-0401-0

**Published:** 2016-07-12

**Authors:** Margarethe E. Wacker, Rudolf A. Jörres, Annika Karch, Armin Koch, Joachim Heinrich, Stefan Karrasch, Holger Schulz, Annette Peters, Sven Gläser, Ralf Ewert, Sebastian E. Baumeister, Claus Vogelmeier, Reiner Leidl, Rolf Holle, for the COSYCONET study group

**Affiliations:** Institute of Health Economics and Health Care Management, Helmholtz Zentrum München (GmbH) - German Research Center for Environmental Health, Comprehensive Pneumology Center Munich (CPC-M), Member of the German Center for Lung Research (DZL), Ingolstädter Landstr. 1, 85764 Neuherberg, Germany; Institute and Outpatient Clinic for Occupational, Social and Environmental Medicine, Ludwig-Maximilians-Universität München, Ziemssenstr. 1, 80336 Munich, Germany; Institute for Biostatistics, Hannover Medical School, Carl-Neuberg-Str. 1, 30625 Hannover, Germany; Institute of Epidemiology I, Helmholtz Zentrum München (GmbH) - German Research Center for Environmental Health, Comprehensive Pneumology Center Munich (CPC-M), Member of the German Center for Lung Research (DZL), Ingolstädter Landstr. 1, 85764 Neuherberg, Germany; Institute of General Practice, University Hospital Klinikum rechts der Isar, Technische Universität München, Orleansstr. 47, 81667 Munich, Germany; Institute of Epidemiology II, Helmholtz Zentrum München (GmbH) - German Research Center for Environmental Health, Ingolstädter Landstr. 1, 85764 Neuherberg, Germany; Department of Internal Medicine B - Cardiology, Intensive Care, Pulmonary Medicine and Infectious Diseases, University Medicine Greifswald, 17475 Greifswald, Germany; Institute for Epidemiology and Preventive Medicine, University of Regensburg, Franz-Josef-Strauß-Allee 11, 93053 Regensburg, Germany; Institute for Community Medicine, University Medicine Greifswald, Walter-Rathenau-Str. 48, 17475 Greifswald, Germany; Department of Medicine, Pulmonary and Critical Care Medicine, University Medical Center Giessen and Marburg, Philipps-University Marburg, Germany, Member of the German Center for Lung Research (DZL), Baldingerstraße, 35043 Marburg, Germany; Institute of Health Economics and Health Care Management, Munich Center of Health Sciences, Ludwig-Maximilians-Universität München, Ludwigstr. 28 / RG, 80539 Munich, Germany

**Keywords:** COPD, Health-related quality of life, Utilities, Cohort study, Comorbidities

## Abstract

**Background:**

Health-related quality of life (HRQL) is an important patient-reported outcome measure used to describe the burden of chronic obstructive pulmonary disease (COPD) which is often accompanied by comorbid conditions.

**Methods:**

Data from 2275 participants in the COPD cohort COSYCONET and from 4505 lung-healthy control subjects from the population-based KORA and SHIP studies were pooled. Main outcomes were the five dimensions of the generic EQ-5D-3 L questionnaire and two EQ-5D index scores using a tariff based on valuations from the general population and an experience-based tariff.

The association of COPD in GOLD grades 1–4 and of several comorbid conditions with the EQ-5D index scores was quantified by multiple linear regression models while adjusting for age, sex, education, body mass index (BMI), and smoking status.

**Results:**

For all dimensions of the EQ-5D, the proportion of participants reporting problems was higher in the COPD group than in control subjects. COPD was associated with significant reductions in the EQ-5D index scores (-0.05 points for COPD grades 1/2, -0.09 for COPD grade 3, -0.18 for COPD grade 4 according to the preference-based utility tariff, all *p* < 0.0001). Adjusted mean index scores were 0.89 in control subjects and 0.85, 0.84, 0.81, and 0.72 in COPD grades 1-4 according to the preference-based utility tariff and 0.76, 0.71, 0.68, 0.64, and 0.58 for control subjects and COPD grades 1-4 for the experience-based tariff respectively. Comorbidities had additive negative effects on the index scores; the effect sizes for comorbidities were comparable to or smaller than the effects of COPD grade 3. No statistically significant interactions between COPD and comorbidities were observed. Score differences between COPD patients and control subjects were most pronounced in younger age groups.

**Conclusions:**

Compared with control subjects, the considerable reduction of HRQL in patients with COPD was mainly due to respiratory limitations, but observed comorbidities added linearly to this effect. Younger COPD patients showed a greater loss of HRQL and may therefore be in specific need of comprehensive disease management.

**Trial registration:**

NCT01245933

## Background

Chronic obstructive pulmonary disease (COPD) is a highly prevalent disease and the fourth leading cause of death worldwide [1]. Therefore, COPD represents a major public health challenge [2]. Patients with COPD are affected to a varying extent by symptoms such as chronic cough, phlegm, dyspnea, and by acute exacerbations or episodes of acute worsening of the respiratory symptoms. These characteristics of COPD are associated with limitations in health-related quality of life (HRQL) [3–7]. Furthermore, HRQL in patients with COPD is also affected by comorbid conditions [8, 9] which are common due to advanced age, common risk factors such as smoking, and the presence of systemic inflammation [10, 11]. Although several studies have demonstrated that comorbidities contribute to low HRQL in patients with COPD [9, 12–14], there is no evidence whether the effect of comorbid conditions on HRQL differs between patients with COPD and individuals without COPD.

Quantifying HRQL is important at the level of individual patients to assess their limitations, the course of disease over time, and the effects of medical interventions. However, HRQL assessment is also essential from a scientific perspective as patient-reported outcomes are important endpoints in clinical studies [15]. For health economic purposes, the effectiveness of health interventions is increasingly quantified using quality-adjusted life years (QALYs) that combine quality and quantity of life into one outcome. Health utilities as a preference-based valuation of health states and a measure of quality can be derived by some generic HRQL instruments such as the EQ-5D questionnaire. These utilities are crucial input parameters for economic COPD models estimating the cost-effectiveness of interventions. The use of national tariffs is recommended for the calculation of utilities whenever possible [16].

This study aims to quantify the impact of COPD on generic HRQL relative to lung-healthy individuals. Therefore, it compares the EQ-5D-3 L questionnaire between a large sample of COPD patients in all grades of airflow limitation and lung-healthy control subjects from two pooled population-based studies. The association of selected comorbid conditions with HRQL is analyzed in COPD patients and control subjects, which allows separation of the effect of COPD on HRQL from the effect of comorbidities. The underlying hypotheses were that 1) HRQL in patients with COPD is impaired compared to control subjects, 2) HRQL deteriorates with increasing COPD grade and 3) comorbidities add to the effect of COPD on HRQL. Finally, national reference values on utilities in different COPD grades are reported that will be useful for economic evaluations of COPD.

## Methods

### Study sample

This is a cross-sectional analysis of three pooled data sources. Data from the baseline visit of the German national COPD cohort COSYCONET (“COPD and Systemic Consequences - Comorbidities Network”) were compared with a lung-healthy control group from the population-based KORA (“Cooperative Health Research in the Augsburg Region”) platform in Southern Germany and the SHIP (“Study of Health in Pomerania”) cohorts in Northern Germany.

The COSYCONET cohort recruited 2741 subjects aged ≥ 40 years with physician-diagnosed COPD between September 2010 and December 2013 through outpatient and inpatient healthcare providers, patient groups, and media campaigns and examined them in 31 study centers all over Germany. Exclusion criteria were previous lung transplantation or lung volume reduction surgery and lung malignancies. All participants had to be clinically stable at their examination visit defined as no moderate or severe exacerbations for at least 4 weeks at the time of enrollment. Details on the cohort have been published previously [17, 18].

The KORA platform comprises population-based health surveys with subsequent follow-up studies in the Augsburg region [19]. Two studies with lung function data were used: Within the KORA F4L study (2010) spirometry was performed in 1051 participants aged 44–65 years. The KORA Age 1 study (2008) recruited participants from previous KORA studies aged 65–89 years and examined lung function by spirometry in a randomly selected sample of 1079 participants [20].

SHIP is a population-based, epidemiological project consisting of two independent cohorts, SHIP and SHIP-TREND, selected from the counties of North- and East of Western Pomerania and the two cities of Greifswald and Stralsund [21]. Data from 1348 participants in the SHIP S2 study (2008–2012) and 2137 SHIP TREND participants (2008–2012) who were aged 40 years or older and performed spirometry were used.

**Fig. 1:**
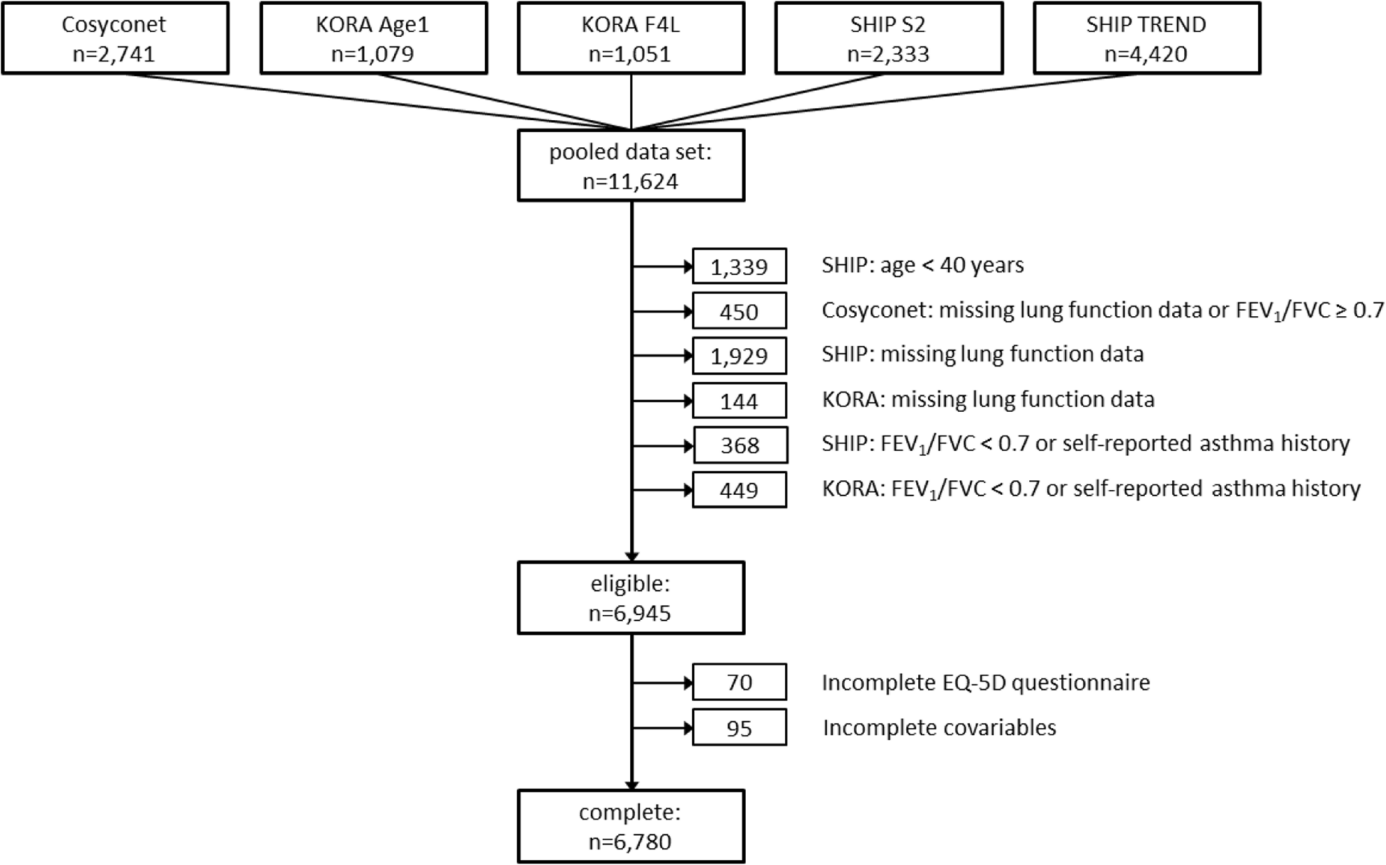
Study flow chart

Figure 1 provides a study flow chart. Pooled data from the KORA Age 1, KORA F4L, SHIP S2 and SHIP-TREND studies covered the general population in an age range of 40–90 years, which is comparable to the COPD cohort.

### Definition of COPD patients and lung-healthy control subjects

In the COSYCONET COPD cohort, standardized spirometry was performed after bronchodilation [17]. In KORA and SHIP, spirometry was performed without bronchodilation. In all studies, COPD was defined as FEV_1_/FVC < 0.7 according to the GOLD criteria [2]. Participants with COPD according to this definition were excluded from the lung-healthy control groups taken from KORA and SHIP, as well as 92 KORA participants and 117 SHIP participants with a self-reported history of asthma.

COPD patients from the COSYCONET cohort were classified as grade 1 with FEV_1_ % pred. ≥ 80, grade 2 with 50 ≤ FEV_1_ % pred. < 80, grade 3 with 30 ≤ FEV_1_ % pred. < 50, and grade 4 with FEV_1_ % pred. < 30. Predicted values were taken from the Global Lung Initiative (GLI) [22]. A total of 430 participants from the cohort reporting physician-diagnosed COPD but with FEV_1_/FVC ≥ 0.7 were excluded from this analysis, as well as 20 participants with incomplete or missing lung function data.

### HRQL assessment

The main outcome of this analysis was the EQ-5D-3 L questionnaire as a generic HRQL instrument that assesses current health status without specifying a recall period. Its descriptive section covers five dimensions of health: mobility, self-care, usual activities, pain/discomfort, and anxiety/depression, with three levels per item (1: no problems, 2: some problems, and 3: extreme problems). The second part of the questionnaire, the valuation section, comprises a Visual Analog Scale (VAS) for valuing health status on a rating scale from 0 (worst imaginable health state) to 100 (best imaginable health state). The VAS was not available in SHIP and KORA.

There are several tariffs for the scoring or valuation of 243 theoretically possible health states as described by the five dimensions of the EQ-5D-3 L questionnaire on a single metric (EQ-5D index) that ranges between 0 and 1 with higher values indicating better health. Country-specific tariffs are preferred to reflect the valuation of health concerns by the specific population. A key aspect of tariffs is whether the valuations come from a representative sample of individuals that values hypothetical health states or from individuals who value their own, current health state [23]. Traditional tariffs are based on the valuation of hypothetical or given health states (GHS) by the general population. They reflect preferences that individuals or society may have for any particular set of health outcomes in a situation of choice and can be interpreted as preference-based utilities. As a broad measure of benefits, utilities are essential for the quality-adjustment of life years and can be used in guiding resource allocation decisions. Alternative methods use the valuation of people who experience an actual health state, referred to as experienced health states (EHS), as these individuals have best insight regarding their health state.

In this analysis, we used the available German time-trade-off tariff of Greiner et al. [24], which is based on the valuation of GHS by a general population sample. The EQ-5D-3 L index resulting from this approach is subsequently referred to as the EQ-5D utility score. As the VAS was not available in all studies, we also used a recently developed German population EQ-5D index from Leidl et al. that is based on EHS [25]. This index uses the VAS for the valuation of health states of the EQ-5D-3 L and is therefore based on the valuation of health states by those who experience this health state instead of the general public. The EQ-5D-3 L index resulting from this additional approach is subsequently referred to as the EQ-5D valuation score.

Over all studies, 69 participants with missing or incomplete EQ-5D data were excluded.

### Covariates and comorbidities

In all studies, participants’ characteristics and data on comorbid conditions were assessed in standardized interviews and questionnaires. Participants’ age and sex were considered as well as their level of school education (basic education ≤9 years, secondary education 10-11 years, higher education > 11 years), body mass index (BMI, kg/m^2^, determined from measured height and weight), and smoking status (smokers were defined as individuals who reported regular or occasional active cigarette smoking within the 4 weeks prior to the interview; former smokers were defined as individuals who reported regular or occasional active cigarette smoking in the past; never smokers were defined as individuals who reported no active cigarette smoking over lifetime). For four COSYCONET participants with missing information on smoking status, former smoking as the most frequent category was assumed. The presence of comorbidities was assessed by asking participants the question: “Has a physician ever diagnosed you with one of the following diseases?”. Information on diabetes, cancer, stroke, myocardial infarction, and arthritis as frequent comorbid conditions was assessed in all studies in a comparable way and considered as comorbidities. Ninety-five observations with missing information regarding covariates were excluded from this analysis.

### Statistical analysis

Characteristics of COPD patients in different grades and of control subjects were compared using analysis of variance (ANOVA) for continuous variables or for unadjusted means of the EQ-5D scores and Chi^2^-tests for categorical variables.

Multiple linear regression models (ordinary least squares, OLS) were conducted to assess the association between COPD in grades 1–4 and the EQ-5D index scores while adjusting for possible confounders.

To quantify the impact of comorbidities on HRQL, all analyses were performed with and without regression adjustment for comorbidities. This approach allows the division of the estimated effect of COPD into a part related to COPD itself and a part related to associated comorbidity. In the first step, the basic models included age group, sex, and level of school education as covariates. Extended models considered smoking status and BMI as possible risk factors and the selected comorbidities as additional covariables.

Interactions between COPD and comorbidities were analyzed by variable selection methods (PROC GLMSELECT with backward selection method) in order to examine non-additive effects of comorbidities on HRQL. In addition, regression models were performed separately for COPD cases and control subjects to investigate possible differences in the effects of comorbidities in each group.

Although numerous studies have based the analysis of EQ-5D index scores on OLS [6, 26, 27], we additionally performed a sensitivity analysis with generalized linear regression models assuming a beta distribution with logit-link [28] in order to account for the skewed and censored distribution of the EQ-5D index scores.

Statistical analyses were performed using SAS software (SAS Institute Inc., Cary, NC, USA, version 9.3), and *p*-values of 0.05 or less were considered to be statistically significant.

## Results

The characteristics of the 2275 participants in the COPD cohort and of 4505 lung-healthy control subjects are shown in Table 1. The COPD cohort had a higher mean age than the pooled control group (65.1 vs. 59.9 years, *p* < 0.0001), a higher proportion of males (61.1 vs. 49.3 %, *p* < 0.0001) and of participants with basic education (55.3 vs. 33.3 %, *p* < 0.0001). The proportion of never smokers was lower in the COPD cohort than in control subjects (6.5 % vs. 42.8 %, *p* < 0.0001) as well as the mean BMI (26.7 vs. 28.4 kg/m^2^, *p* < 0.0001). Regarding comorbidities, stroke, cancer, and myocardial infarction were reported significantly more often in the COPD cohort than in the control group.

**Table 1:** Characteristics of the study population

	KORA lung- healthy control subjects	SHIP lung-healthy control subjects	Pooled control group	COPD grade 1	COPD grade 2	COPD grade 3	COPD grade 4	Total COPD cohort	*p-*value (total cohort vs. pooled control subjects)
n	1,506	2,999	4,505	204	954	869	248	2,275	
age (years)	63.4 (11.8)	58.1 (10.8)	59.9 (11.4)	66.1 (8.7)	65.7 (8.5)	65.0 (8.2)	62.1 (8.0)	65.1 (8.4)	*<0.0001* ^a^
% age < 55 years	28.0 (421)	39.7 (1,190)	35.8 (1,611)	9.8 (20)	10.2 (97)	11.1 (96)	18.2 (45)	11.3 (258)	*<0.0001* ^b^
% age 55-64 years	28.0 (421)	28.8 (863)	28.5 (1,284)	27.5 (56)	31.9 (304)	34.4 (299)	43.2 (107)	33.7 (766)	
% age 65-74 years	21.4 (322)	24.5 (734)	23.4 (1,056)	48.0 (98)	44.6 (425)	44.0 (382)	31.9 (79)	43.3 (984)	
% age > 74 years	22.7 (342)	7.1 (212)	12.3 (554)	14.7 (30)	13.4 (128)	10.6 (92)	6.9 (17)	11.7 (267)	
% males	48.1 (724)	49.9 (1,496)	49.3	60.3 (123)	60.6 (578)	61.1 (531)	64.1 (159)	61.1	*<0.0001* ^b^
FEV_1_ (liter)	3.06 (0.9)	3.16 (0.8)	3.13 (0.8)	2.62 (0.6)	1.85 (0.5)	1.20 (0.3)	0.75 (0.2)	1.55 (0.6)	*<0.0001* ^a^
FVC (liter)	3.92 (1.1)	3.96 (1.0)	3.95 (1.1)	4.11 (0.9)	3.31 (0.9)	2.67 (0.8)	2.05 (0.6)	3.00 (1.0)	*<0.0001* ^a^
% basic education (≤9 school years)	56.2 (847)	21.8 (854)	33.3 (1,501)	48.0 (98)	52.1 (497)	60.0 (521)	57.7 (143)	55.3 (1,259)	*<0.0001* ^b^
% secondary education (10–11 school years)	22.7 (342)	52.8 (1,584)	42.8 (1,926)	25.5 (52)	28.6 (273)	25.2 (219)	29.4 (73)	27.1 (617)	
% higher education (>11 school years)	21.1 (317)	25.4 (761)	23.9 (1,078)	26.5 (54)	19.3 (184)	14.8 (129)	12.9 (32)	17.5 (399)	
% smoker	11.3 (170)	16.1 (484)	14.5 (654)	29.9 (61)	28.9 (276)	21.8 (189)	14.5 (36)	24.7 (562)	*<0.0001* ^b^
% former smoker	39.2 (590)	44.4 (1,332)	42.7 (1,922)	62.8 (128)	63.7 (608)	72.6 (631)	80.2 (199)	68.8 (1,566)	
% never smoker	49.5 (746)	39.5 (1,183)	42.8 (1,929)	7.4 (15)	7.3 (70)	5.6 (49)	5.2 (13)	6.5 (147)	
BMI (kg/m^2^)	28.2 (4.7)	28.6 (4.7)	28.4 (4.7)	26.6 (4.7)	27.5 (5.1)	26.4 (5.4)	24.4 (5.0)	26.7 (5.2)	*<0.0001* ^a^
% normal weight (18.5 ≤ BMI < 25)	24.4 (368)	22.7 (681)	23.3 (1,049)	36.8 (75)	32.5 (310)	38.4 (334)	50.0 (124)	37.1 (843)	*<0.0001* ^b^
% overweight (25 ≤ BMI < 30)	45.8 (690)	43.8 (1,313)	44.5 (2,003)	41.7 (85)	39.0 (372)	36.1 (314)	28.6 (71)	37.0 (842)	
% obese (BMI ≥ 30)	29.6 (445)	33.4 (1,003)	32.1 (1,448)	20.1 (41)	27.0 (258)	20.9 (182)	11.7 (29)	22.4 (510)	
% underweight (BMI < 18.5)	0.2 (3)	0.1 (2)	0.1 (5)	1.5 (3)	1.5 (14)	4.5 (39)	9.7 (24)	3.5 (80)	
% diabetes	10.4 (156)	13.3 (399)	12.3 (555)	10.3 (21)	13.3 (127)	12.8 (111)	11.3 (28)	12.6 (287)	*0.73* ^b^
% stroke	4.1 (61)	2.5 (75)	3.0 (136)	3.9 (8)	4.3 (41)	4.8 (42)	2.4 (6)	4.3 (97)	*0.008* ^b^
% myocardial infarction	5.1 (76)	3.5 (106)	4.0 (182)	6.4 (13)	8.8 (84)	9.6 (83)	5.2 (13)	8.5 (193)	*<0.0001* ^b^
% cancer	9.5 (143)	8.1 (242)	8.6 (385)	11.8 (24)	12.2 (116)	10.1 (88)	7.3 (18)	10.8 (246)	*0.002* ^b^
% arthritis	12.4 (186)	5.6 (168)	7.9 (354)	10.3 (21)	8.1 (77)	8.6 (75)	4.8 (12)	8.1 (185)	*0.69* ^b^
EQ-5D utility score	0.90 (0.14)	0.90 (0.14)	0.90 (0.14)	0.85 (0.18)	0.84 (0.19)	0.81 (0.21)	0.74 (0.24)	0.82 (0.21)	*<0.0001* ^a^
EQ-5D valuation score	0.76 (0.14)	0.77 (0.13)	0.77 (0.13)	0.71 (0.14)	0.68 (0.16)	0.64 (0.17)	0.58 (0.17)	0.66 (0.17)	*<0.0001* ^a^

Data are mean (standard deviation) or percentage

^a^ *p*-value based on ANOVA^b^ *p*-value based on Chi^2^-test

**Fig. 2:**
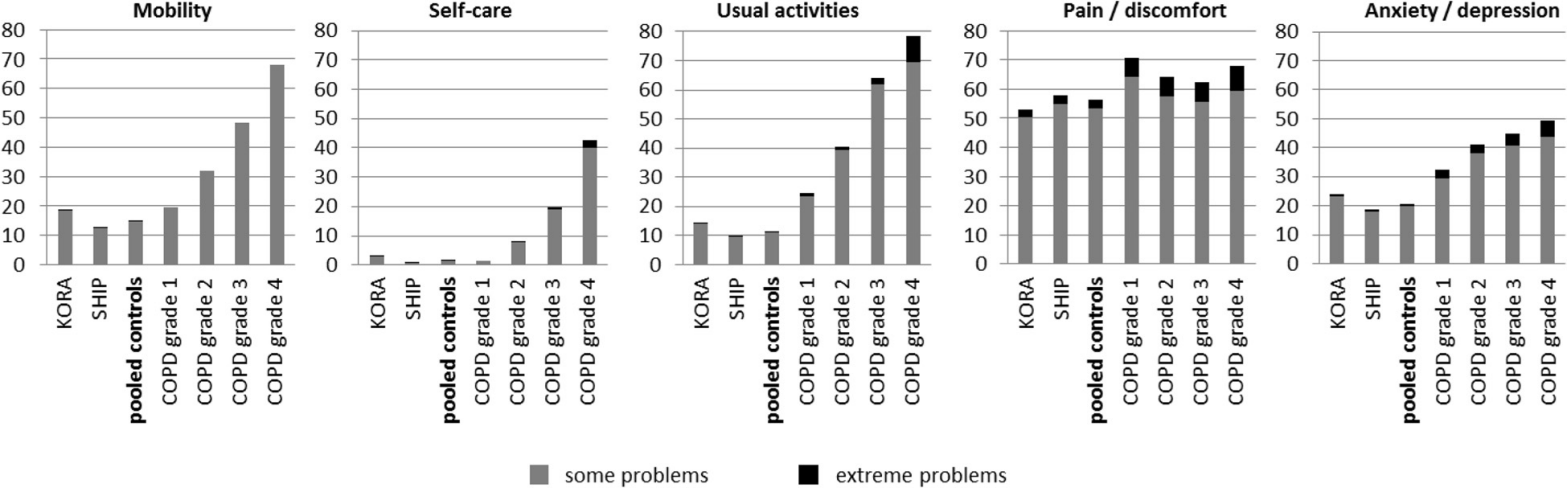
EQ-5D dimensions: % of participants reporting problems (unadjusted)

For the EQ-5D dimensions mobility, usual activities, and anxiety/depression, the proportion of participants reporting some or extreme problems was higher in the COPD groups than in control subjects and increased with higher COPD grade, as illustrated in Fig. 2. Especially for mobility and usual activities, a large proportion of COPD patients reported some or extreme problems, with up to 79 % of participants in COPD grade 4 for usual activities. Regarding self-care, the proportion of participants reporting problems was comparable in COPD grade 1 and the control group, but steadily increased from grade 2 to grade 4 in the COPD group. As to pain and discomfort, a slightly higher proportion of COPD patients reported problems compared with control subjects, but within the COPD group, proportions were comparable with 70.6 % in grade 1 and 64 % in grade 3.

Unadjusted mean EQ-5D utility and valuation scores for COPD grades 1–4 were lower than in the control group and decreased with increasing disease severity (Table 1).

Table 2 shows the results of the regression analyses. After adjusting for differences in age, sex, and education between groups, all COPD grades were associated with statistically significant reductions in both index scores. This reduction increased with higher disease grade. The effect estimates of COPD grades 1 and 2 were of a similar magnitude when considering the utility score and differed slightly when considering the valuation score. When controlling for smoking status, weight, and comorbidities in extended regression models, the negative effects of COPD grades 1–4 remained stable. All comorbid conditions had additional negative effects on the index scores; these effects were additive as all interaction terms between COPD grade and comorbid conditions were not statistically significant (results not shown). The effect sizes for comorbidities were comparable to or smaller than the effects of COPD grade 3, whereby a history of arthritis or stroke had the largest effects. The effect estimate of obesity was similar to the estimate of COPD grades 1 or 2.

**Table 2:** Results of multiple linear regression models

		EQ-5D utility score		EQ-5D valuation score	
Covariate		Basic model	Extended model	Basic model	Extended model
Intercept		**0.91** [0.90 to 0.92]	**0.96** [0.94 to 0.97]	**0.78** [0.77 to 0.79]	**0.82** [0.81 to 0.83]
**COPD**	no COPD	ref.	ref.	ref.	ref.
	**grade 1**	**−0.04** [-0.06 to -0.02]	**−0.05** [-0.07 to -0.02]	**−0.05** [-0.07 to -0.03]	**−0.06** [-0.07 to -0.04]
	**grade 2**	**−0.05** [-0.06 to -0.04]	**−0.05** [-0.06 to -0.04]	**−0.08** [-0.09 to -0.07]	**−0.08** [-0.09 to -0.07]
	**grade 3**	**−0.08** [-0.09 to -0.07]	**−0.09** [-0.10 to -0.08]	**−0.12** [-0.13 to -0.11]	**−0.13** [-0.14 to -0.12]
	**grade 4**	**−0.16** [-0.18 to -0.14]	**−0.18** [-0.20 to -0.15]	**−0.18** [-0.20 to -0.16]	**−0.20** [-0.21 to -0.18]
Age (years)	< 55	ref.	ref.	ref.	ref.
	55-64	**−0.02** [-0.04 to -0.01]	−0.01 [-0.02 to 0.00]	**−0.03** [-0.04 to -0.02]	**−0.02** [-0.02 to -0.01]
	65-74	**−0.02** [-0.03 to -0.01]	−0.01 [-0.02 to 0.00]	**−0.01** [-0.02 to -0.00]	0.00 [-0.01 to 0.01]
	> 74	**−0.06** [-0.07 to -0.04]	**−0.04** [-0.05 to -0.02]	**−0.06** [-0.07 to -0.04]	**−0.04** [-0.05 to -0.02]
Sex	male	ref.	ref.	ref.	ref.
	female	**−0.01** [-0.02 to -0.01]	**−0.02** [-0.03 to -0.01]	**−0.02** [-0.03 to -0.02]	**−0.03** [-0.04 to -0.02]
Education	basic (≤ 9 school years)	ref.	ref.	ref.	ref.
	secondary (10-11 school years)	**0.01** [0.01 to 0.02]	0.01 [-0.00 to 0.02]	**0.02** [0.01 to 0.02]	**0.01** [0.00 to 0.02]
	higher (>11 school years)	**0.04** [0.03 to 0.05]	**0.03** [0.02 to 0.04]	**0.04** [0.03 to 0.05]	**0.03** [0.02 to 0.04]
Smoking status	never smoker		ref.		ref.
	smoker		**−0.02** [-0.03 to -0.00]		**−0.01** [-0.02 to -0.00]
	former smoker		**−0.01** [-0.02 to -0.00]		−0.01 [-0.01 to 0.00]
Weight	normal weight (18.5 ≤ BMI < 25)		ref.		ref.
	underweight (BMI < 18.5)		0.00 [-0.03 to 0.04]		0.01 [-0.02 to 0.04]
	overweight (25 ≤ BMI < 30)		**−0.02** [-0.03 to -0.01]		**−0.02** [-0.02 to -0.01]
	obese (BMI ≥ 30)		**−0.05** [-0.06 to -0.04]		**−0.05** [-0.06 to -0.04]
Comorbidities	diabetes		**−0.03** [-0.04 to -0.02]		**−0.02** [-0.04 to -0.01]
	stroke		**−0.05** [-0.07 to -0.03]		**−0.05** [-0.07 to -0.03]
	myocardial infarction		**−0.04** [-0.05 to -0.02]		**−0.04** [-0.05 to -0.02]
	cancer		**−0.02** [-0.04 to -0.01]		**−0.02** [-0.03 to -0.01]
	arthritis		**−0.08** [-0.10 to -0.07]		**−0.08** [-0.09 to -0.07]

Estimates with *p* < 0.05 are printed in bold

**Fig. 3:**
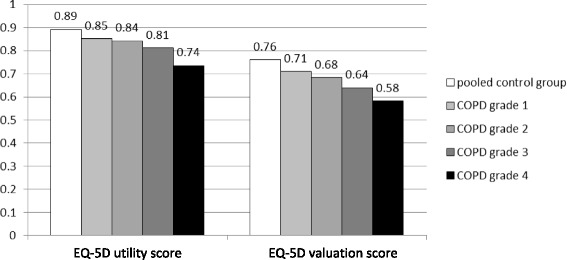
Adjusted mean EQ-5D index scores. Models adjusted for age, sex, and education

**Fig. 4:**
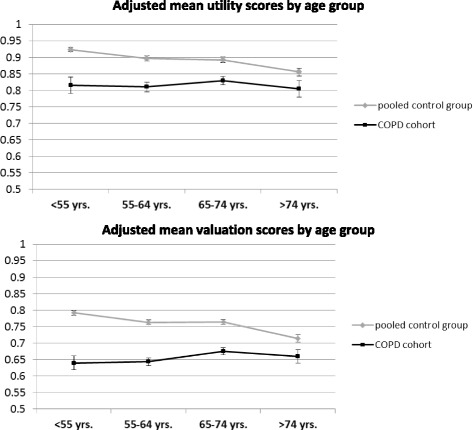
Adjusted EQ-5D index score stratified by age group. Models adjusted for age, sex, and education

Table 3 shows the effect estimates of comorbid conditions, stratified for the COPD cohort and the control group. Most of the estimates were comparable in cases and control subjects. Figure 3 shows the adjusted mean index scores for all groups resulting from the basic regression model. Figure 4 illustrates adjusted mean index scores by age group. For both index scores, the gap between COPD patients and control subjects was more pronounced in younger than in older age groups.

**Table 3:** Association of comorbid conditions with the EQ-5D index scores stratified for COPD cases and control subjects

	EQ-5D utility score		EQ-5D valuation score	
Covariate	COPD cases	Control subjects	COPD cases	Control subjects
Diabetes	**−0.03** [-0.05 to -0.00]	**−0.03** [-0.05 to -0.02]	−0.01 [-0.03 to 0.01]	**−0.03** [-0.05 to -0.02]
Stroke	−0.04 [-0.08 to 0.00]	**−0.05** [-0.08 to -0.03]	−0.03 [-0.06 to 0.00]	**−0.06** [-0.08 to -0.04]
Myocardial infarction	**−0.05** [-0.08 to -0.02]	**−0.02** [-0.04 to -0.00]	**−0.05** [-0.07 to -0.02]	**−0.03** [-0.05 to -0.01]
Cancer	**−0.03** [-0.05 to -0.00]	**−0.02** [-0.03 to -0.00]	−0.02 [-0.04 to 0.00]	**−0.03** [-0.04 to -0.01]
Arthritis	**−0.09** [-0.12 to -0.06]	**−0.08** [-0.09 to -0.06]	**−0.07** [-0.10 to -0.05]	**−0.08** [-0.09 to -0.06]
Obesity (BMI ≥ 30)	**−0.06** [-0.08 to -0.04]	**−0.04** [-0.05 to -0.03]	**−0.04** [-0.06 to -0.02]	**−0.04** [-0.05 to -0.05]

Estimates with *p* < 0.05 are printed in bold

Models adjusted for age, sex, education, BMI class, and smoking status

The adjusted mean index scores based on a model assuming a beta-distribution with logit-link differed only marginally from those of the OLS models: a difference of -0.01 to +0.03 was observed for the means of the groups according to utility score and -0.01 to +0.01 according to the valuation score.

## Discussion

Based on a large pooled data set, this study corroborates the significant reduction of generic HRQL in patients with COPD compared with lung-healthy control subjects. HRQL limitations increased with higher COPD disease grade. The coexistence of COPD with other diseases contributes to the overall burden by increasing HRQL limitations. Therefore, comorbid conditions are important to assess in the context of COPD management. The novel finding of this study was that the HRQL reductions were found to be primarily due to the lung disorder, whereas the effects of comorbidities were weaker and additive. Comorbid arthritis, stroke, and obesity had the largest additional effects on the HRQL index scores. However, significant interactions between COPD grades and comorbidities were not found. Notably, the HRQL gap between COPD patients and control subjects was larger in younger than in older age groups, which could be explained by a higher impact of dyspnea on HRQL [29]. As an individualized, patient-centered approach is postulated as the ideal care of COPD patients [30], younger patients with COPD may be an important target group for specific disease management.

The EQ-5D questionnaire is the most frequently used instrument for the calculation of health utilities and recommended by several reimbursement and academic authorities worldwide [31]. The validity, reliability, responsiveness, and discriminative properties of the EQ-5D in populations with COPD have been examined in several studies [5, 12, 32–34]. For the EQ-5D index scores used in this study, a minimal clinically important difference (MCID) has not yet been established. Based on studies using other scoring algorithms and in diseases other than COPD, it may range between 0.04 and 0.07 [35, 36]. Therefore, the effects for COPD grades observed in this study can be seen as clinically relevant.

This evidence corroborates previous studies that have already shown that increasing disease severity in COPD is associated with a reduction in EQ-5D utility and other generic or disease-specific HRQL scores [37–40]. However, it also expands previous evidence as there is a lack of studies considering the entire spectrum of disease grades and a comparison with control subjects [6, 12, 39]. The variation in published mean EQ-5D utility scores per COPD grade is considerable. Besides diverging underlying populations and definitions of COPD, the comparability of utilities with previous studies is hampered by differences in the scoring algorithms for the EQ-5D index. Differences between national EQ-5D tariffs have been shown to be substantial because of different methodological approaches, but also, to a minor degree, due to cultural differences in valuation. As these differences may influence the results of cost-utility analyses [16], national data on utilities are required for economic evaluations of COPD in Germany.

Einarson et al. reviewed 44 original articles that reported utilities related to COPD [41]. Irrespective of disease classification and utility instrument, methods or tariffs used, mean utilities were 0.83, 0.77, 0.71, and 0.61 for GOLD grades 1–4 respectively. A review by Pickard et al. reported pooled mean utility scores based on a UK scoring algorithm of 0.74, 0.74, 0.69, and 0.61 for GOLD grades 1–4 [40]. Especially for early COPD grades the pooled mean utilities reported by Pickard et al. were lower than those found in this analysis and those reported by Einarson et al. Similar to Pickard et al., our study found comparable mean utilities for GOLD grades 1 and 2. This finding might be explained by possible ceiling effects of the EQ-5D questionnaire that limit the ability of this instrument to discriminate well between early COPD grades and have been described previously [6, 12, 34]. Nevertheless, the availability of control subjects enabled us to show a reduction in the EQ-5D index scores even in early disease grades.

There are only a few studies that have considered the effect of comorbidities on utilities in COPD patients. Most of them used the number of comorbid conditions or a weighted comorbidity index and found that a higher number of comorbidities is associated with a reduced utility [12, 14, 34]. By pooling data from patients with COPD and lung-healthy control subjects, we were able to separate the effect of COPD on HRQL from the effect of comorbidities. Our results show that the effects of comorbid conditions on the EQ-5D index scores were comparable between patients with COPD and lung-healthy control subjects and the size of comorbid effects was equal to or even smaller than the impact of COPD grade 3. Arthritis and stroke showed the highest effects and are therefore in need of special consideration. In line with our results, a previous study also did not find interactions or effect-modifications between COPD disease severity and comorbidity [12]. However, a comparison with control subjects was not considered.

The main strength of this study lies in the large sample size covering all COPD grades and the availability of control subjects from two population-based cohort studies. Individual data with standardized variable definitions could be pooled for this analysis.

Besides the established German EQ-5D utility tariff [24], an experience-based tariff for the valuation of health states into a single index score was additionally considered. Although national utility scores are needed for the calculation of QALYs and for cost-utility analyses in COPD, experience-based scores may be important for decision makers who want to base their decisions on experienced health states rather than on the valuation of these health states by the general population [25, 42]. Whereas the utility tariff includes preferences on hypothetical health states, the valuation by the experience-based tariff refers to the health state of respondents, and valuation is done by VAS [25]. Both differences may contribute to the finding that valuations of the experience-based index are lower than those of the utility-tariff. As shown by another study of the COSYCONET cohort, preference-based utilities and patients’ VAS valuations may also differ: Between grade 1 and 4, the utility-tariff indicated a difference of 0.13 while for patients’ valuations, this was 0.22 [34], thus making the choice of valuation used in this study important to the results obtained.

This study is subject to methodologic limitations. First, the possibility of selection bias in all studies considered might have led to an over- or underestimation of the effect of COPD on the index scores. Although the results are based on data from a large COPD cohort that may be more easily generalized than those of clinical studies, as well as on a pooled control group from northern and southern Germany, it cannot be excluded that they are not fully representative for the entire German population. Second, the necessity of standardized and comparable covariates throughout all studies limited the range of variables that could have been taken into account. In particular, a comparison of the influence of further comorbidities was limited by the lack of comparable information. Therefore, the reductions in HRQL in COPD grades might be explained partly by the occurrence of other comorbidities that could not be compared between COPD cases and control subjects. This restriction could have led to an overestimation of the effects of COPD on index scores. Third, the comorbidities considered in our study may reflect historic conditions, such as infarction or cancer. The effects of recent comorbid events on HRQL might be more significant. Finally, the use of pre-bronchodilator spirometry data to exclude subjects with COPD from the control group might have led to a misclassification of subjects with asthma as COPD patients. Therefore, subjects with FEV_1_/FVC ≥ 0.7 and self-reported asthma were additionally excluded in order to create a lung-healthy control group. This approach might have led to an overestimation of the effects of COPD on HRQL scores because our control group might be healthier than a control group excluding COPD patients only.

## Conclusions

Compared with control subjects, generic HRQL is considerably impaired in patients with COPD, even in early disease grades, and this was primarily caused by the lung disease and not the comorbidities. As this impairment is more pronounced in younger age groups, these patients are a particularly relevant target group for specific or comprehensive disease management.

## Abbreviations

ANOVA, analysis of variance; BMI, body mass index; COPD, chronic obstructive pulmonary disease; COSYCONET, COPD and systemic consequences - comorbidities network; EQ-5D-3 L, EuroQol 5 dimensions questionnaire with 3 levels; FEV_1_, forced expiratory volume in 1 second; FVC, forced vital capacity; HRQL, health-related quality of life; KORA, Cooperative Health Research in the Augsburg Region; MCID, minimum clinically important difference; OLS, ordinary least squares, QALY, quality-adjusted life year; SD, standard deviation; SHIP, Study of Health in Pomerania, VAS, visual analog scale
